# Special Issue “Next-Generation Technologies to Understand Mechanisms of Virus Infections”

**DOI:** 10.3390/v15010033

**Published:** 2022-12-22

**Authors:** Allan R. Brasier

**Affiliations:** Institute for Clinical and Translational Research, University of Wisconsin-Madison, Madison, WI 53705, USA; abrasier@wisc.edu

RNA viruses are responsible for substantial morbidity and health burden. Some 200 diverse human-transmissible RNA viruses are known, including respiratory syncytial virus (RSV), influenza virus, measles virus, HCV, HIV, dengue, rotavirus, and others that produce substantial disease burden. These viruses exhibit substantial mutability, leading to antigenic variation, enhanced virulence, changed cellular tropism and expanded host range. This feature results in RNA viruses representing current emerging zoonotic transmissions to humans, including SARS-CoV2, MERS and Ebola. Over the last three years, we have witnessed the morbidity, mortality and economic impact of these zoonotic RNA virus infections that have become a major focus of therapeutics and vaccine development efforts.

Despite intensive study, the mechanisms of host–viral interactions are still incompletely understood. Work published elsewhere in this journal has documented that RNA virus replication triggers innate responses, activates unfolded protein responses, and produces metabolic adaptations. The variability in host responses affects the dynamics of the innate immune response, viral clearance and establishment of adaptive immunity, resulting in distinct manifestations of disease and/or chronic sequelae. Viral mutations influence fitness, spread and ability to modify host innate responses. 

Next-generation technologies coupled with bioinformatics analyses provide global, novel insights into the complexity of virus–host interactions. These rapidly emerging technologies include next-generation sequencing (NGS), proteomics, epigenetics, metabolomics and their integration. Especially exciting and germane to the COVID-19 pandemic are the computational approaches to viral replication, fitness and spread. These approaches provide substantial opportunities to advance the understanding of disease mechanisms, improve detection and advance therapeutics for RNA virus infections. 

This Special Issue was conceptualized as a home for describing emerging methods and findings using next-generation technologies. We have reviewed and published 11 exciting contributions to this topic that broadly advance this field, highlighted below.

This Special Edition contains papers describing NGS approaches, including clinical applications in HIV, HCV and HPV. In particular, Zucko et al. [1] describe the measurement of circRNA, RNA arising from mRNA splicing, in HIV-infected patients treated with antiretroviral therapy. This circRNA NGS analysis found that antiretroviral therapy may cause a genetic diversification of peripheral viral reservoirs. Separately, Scutari et al. [2] used NGS to examine whether treatment interruption influenced residual viremia and resistance mutations in a pilot study of HIV-infected patients. Additionally, Minosse et al. [3] describe an approach to investigate HCV recurrence in infected patients that sustain a therapeutic response. In the rare situations of treatment failure in HCV infection, it is difficult to distinguish between relapse and reinfection; this work shows how NGS can be used to identify the mechanism of recurrence.

Single-molecule, real-time (SMRT) sequencing is an exciting new technology that provides an unbiased understanding of mRNA splicing. However, it is difficult to quantitate the changes in the transcripts. In this issue, Xu et al. [4] demonstrate that SMRT sequencing can be combined with short-read mRNA reads to quantitate changes in RNA processing in RSV-infected epithelial cells. These investigators found that RSV has a substantial impact on exon occlusion, intron inclusion, and alternative transcription start site utilization, including those encoding key innate regulators. 

Next-generation live-cell imaging technologies with DNA fluorescent dyes can provide valuable information about viral spreading behavior in near real-time. Arias-Arias et al. [5] demonstrated a live-cell fluorescent imaging application that improves upon conventional plaque assays in real-time and at the single-cell level. 

The field of proteomics is rapidly advancing to understand dynamic changes in protein–protein interactions in response to viral infections. Mann et al. [6] demonstrate the application of native immunoprecipitation and a highly sensitive Parallel Accumulation–Serial Fragmentation mass spectrometry to understand the dynamic changes in the protein–protein interaction network for a key epigenetic regulator, BRD4, in RSV-infected epithelial cells. The role of acetylated lysine binding was extended using a small molecule probe to disrupt BRD4′s acetyl-lysine binding pocket. This group observed that the BRD4 inhibitors disrupted binding to acetylated transcriptional regulators. This study is complemented by a review by Simanjuntak et al. [7] that describes novel MS tools to study the structure and composition of protein complexes, providing critical mechanistic insights into their functions. New approaches in top-down and bottom-up mass spectrometry-based are presented to identify novel- and tractable antiviral targets.

Advances in functional metabolomics have been presented for the analysis of the innate responses to RSV infection. Specifically, Connelly et al. [8] describe the application of functional metabolomics, metabolic flux analysis and NGS of nasal airway epithelial cells from RSV-infected infants in the first year of life. This group found evidence for a significant increase in glucose metabolism and significant sexual dimorphism that was most pronounced in males. 

The computational modeling of viral replication provides profound insight into replicative viral advantage. Using this approach, Grabowski et al. [9] estimated the growth rates of expanding mutations acquired by the SARS-CoV2 variant of concern to identify the competitive advantage of a viral substrain that compromises neutralizing antibody binding. 

Nanostring is a robust and reproducible technology that can detect nanoscale amounts of RNA. Rajeevan et al. [10] describe the application of Nanostring technology for high throughput HPV testing of multiple HPV types that will enable HPV vaccination studies. 

Human Noroviruses (HuNoVs) are low abundant and fastidious viruses that are difficult to culture and diagnose. In this Special Edition, Zhang et al. [11] describe how transposase-assisted RNA/DNA hybrid Co-tagmentation can be used in a library preparation of HuNoVs for NGS detection in a complex background.

Please join me in thanking the contributors and the Editors and Editorial staff for assembling this Special Issue. We look forward to furthering the applications and insights that these technologies will bring to the field.
